# The incidence of all‐cause dementia and Alzheimer’s disease from around the world: data from the COSMIC collaboration

**DOI:** 10.1002/alz.087341

**Published:** 2025-01-09

**Authors:** Ashleigh S. Vella, Rachel Visontay, Darren M. Lipnicki, Emma Nichols, Jamie Steinmetz, Richard B. Lipton, Juan J. Llibre‐Rodriguez, Mindy J. Katz, Nikolaos Scarmeas, Maëlenn Guerchet, Pierre‐Marie Preux, Karen Ritchie, Isabelle Carriere, Maria Skaalum Petersen, Ingmar Skoog, Elena Rolandi, Ki Woong Kim, Steffi G. Riedel‐Heller, Suzana Shahar, Mary Ganguli, Kaarin J. Anstey, Antonio Lobo, John D. Crawford, Louise Mewton, Perminder S. Sachdev, other COSMIC authors

**Affiliations:** ^1^ Centre for Healthy Brain Ageing, University of New South Wales, Sydney, NSW Australia; ^2^ The Matilda Centre for Research in Mental Health and Substance Use, The University of Sydney, Sydney, NSW Australia; ^3^ Centre for Healthy Brain Ageing, Discipline of Psychiatry & Mental Health, School of Clinical Medicine, University of New South Wales, Sydney, NSW Australia; ^4^ Institute for Health Metrics and Evaluation, Seattle, WA USA; ^5^ University of Southern California, Los Angeles, CA USA; ^6^ Institute for Health Metrics and Evaluation, University of Washington, Seattle, WA USA; ^7^ Department of Neurology, Albert Einstein College of Medicine, Bronx, NY USA; ^8^ Albert Einstein College of Medicine, Bronx, NY USA; ^9^ Dementia Research Unit/Medical University of Havana, Havana, Havana Cuba; ^10^ Columbia University, New York, NY USA; ^11^ National and Kapodistrian University of Athens, Athens Greece; ^12^ National Institute for Health and Medical Research U1094, Institut de Recherche pour le Developpement UMR270, Epidemiology of Chronic Diseases in Tropical Zone, Institute of Epidemiology and Tropical Neurology, OmegaHealth, University Limoges, Centre Hosp, Limoges France; ^13^ Institut du Cerveau Trocadéro, Paris France; ^14^ Institut for Neurosciences of Montpellier, University Montpellier, National Institute for Health and Medical Research, Montpellier France; ^15^ Institute for Neurosciences of Montpellier, University of Montpellier, INSERM, Montpellier France; ^16^ Institut du Cerveau Trocadéro, Pa France; ^17^ The National Hospital of Faroe Island, Tórshavn Faroe Islands; ^18^ Psychiatry, Cognition and Old Age Psychiatry, Sahlgrenska University Hospital, Gothenburg Sweden; ^19^ Neuropsychiatric Epidemiology, Institute of Neuroscience and Physiology, Sahlgrenska Academy, University of Gothenburg, Mölndal Sweden; ^20^ Golgi Cenci Foundation, Abbiategrasso Italy; ^21^ University of Pavia, Pavia Italy; ^22^ Seoul National University College of Medicine, Seoul Korea, Republic of (South); ^23^ Seoul National University Bundang Hospital, Seongnam Korea, Republic of (South); ^24^ Seoul National University College of Natural Sciences, Seoul Korea, Republic of (South); ^25^ Institute of Social Medicine, Occupational Health and Public Health (ISAP), Medical Faculty, University of Leipzig, Leipzig Germany; ^26^ Community Rehabilitation and Aging Research Centre, Faculty of Health Sciences, Universiti Kebangsaan Malaysia, Kajang Selangor Malaysia; ^27^ Division of Geriatrics & Neuropsychiatry, Department of Psychiatry, University of Pittsburgh, Pittsburgh, PA USA; ^28^ University of New South Wales, Sydney, NSW Australia; ^29^ Neuroscience Research Australia, Sydney, NSW Australia; ^30^ UNSW Ageing Futures Institute, Sydney, NSW Australia; ^31^ University of Zaragoza, Zaragoza Spain; ^32^ Instituto de Investigación Sanitaria Aragón, Zaragoza Spain; ^33^ Centro de Investigación Biomédica en Red de Salud Mental, Madrid Spain; ^34^ Centre for Healthy Brain Ageing (CHeBA), UNSW Sydney, Sydney, NSW Australia; ^35^ The Matilda Centre for Research in Mental Health and Substance Use, The University of Sydney, Sydney, NSW Australia; ^36^ Neuropsychiatric Institute, Prince of Wales Hospital, Randwick, NSW Australia; ^37^ Centre for Healthy Brain Ageing (CHeBA), University of New South Wales, UNSW Sydney, NSW Australia

## Abstract

**Background:**

High‐income countries (HICs) are over‐represented in current global dementia incidence rates, skewing estimates. Variance in diagnostic methods between HICs and low‐ and middle‐income countries (LMICs) is speculated to contribute to the regional differences in rates. Cohort Studies of Memory in an International Consortium (COSMIC) offers a unique opportunity to address these research inequalities by harmonising data from international studies, including representation from LMICs. This study aimed to identify dementia incidence rates by age and sex in various regions worldwide, where data for dementia diagnosis were available.

**Method:**

Data were obtained from 36 members of COSMIC, representing 28 countries across 6 continents (HICs: Australia, Canada, Faroe Islands, France, Germany, Greece, Italy, Japan, Netherlands, South Korea, Spain, Sweden, & USA; LMICs: Brazil, China, Cuba, Dominican Republic, Ecuador, Indonesia, Malaysia, Mexico, Nigeria, Peru, Philippines, Republic of Congo, & Tanzania). For each member study, we calculated incidence rates for all‐cause dementia. Findings from 14 studies, with a consensus diagnosis are presented in the results. Using an Item Response Theory approach, we are currently calculating a comparable incidence rate for those studies without a consensus diagnosis.

**Result:**

Consistent with previous trends, incidence rates (per 100 person‐years) increased with age, from 65‐70 years‐old to 85‐90 years‐old, for both males (i.e., Republic of Congo, 4.41 to 19.57; France, 0.46 to 3.89; USA, 0.17 to 3.22; Spain, 0.31 to 4.22; 65‐70 & 85‐90 cohorts respectively) and females (i.e., Republic of Congo, 3.57 to 15.31; France, 0.45 to 3.72; USA, 0.22 to 4.25; Spain, 0.36 to 4.96; 65‐70 & 85‐90 cohorts respectively). There were no sex differences in incidence rates in younger age groups (60‐65). Among older age groups, however, women tended to have higher incidence rates than men, in some countries (Faroe Islands, Germany, Sweden, and USA).

**Conclusion:**

Geographical differences in dementia incidence rates likely represent inherent variation among countries, beyond methodological considerations. We are working to expand the range of studies and regions for which we calculate dementia incidence rates. This involves the development of approaches to classify and harmonise incident dementia in studies lacking consensus diagnoses. Doing so will bolster LMIC representation.

